# Eosinophilic Esophagitis: Current Aspects of a Recently Recognized Disease

**DOI:** 10.4021/gr2010.04.201w

**Published:** 2010-03-20

**Authors:** Alfredo J. Lucendo, Sonia Gonzalez-Castillo, Danila Guagnozzi, Jose Luis Yague-Compadre, Angel Arias

**Affiliations:** a Department of Gastroenterology, Hospital General de Tomelloso, Tomelloso, Spain; b Department of Pathology and ^c^Research Unit, Complejo Hospitalario Mancha Centro, Alcazar de San Juan, Spain

**Keywords:** Eosinophilic esophagitis, Fluticasone, Endoscopic dilation, Dietary treatment

## Abstract

Eosinophilic esophagitis (EoE) is a chronic clinicopathological entity characterized by large numbers of intraepithelial eosinophils infiltrating the esophageal mucosa. The inflammation leads to alterations in the caliber and the motility of the organ, which determines esophageal symptoms, especially dysphagia and frequent food impaction. Firstly described in 1978, EoE represents today an increasingly recognized disease, with cases coming from all developed countries and rising epidemiology. The origin of EoE has been related to allergy to food components or inhalants, and a number of studies support a Th2-type reaction in the origin of the disease. Thus, several treatment strategies based on controlling the exposition to triggering allergens or therapies using anti-allergic drugs have demonstrated efficacy in EoE. Since EoE frequently presents with esophageal stenosis, endoscopic dilation has been also used in treating these patients, but a high risk of complications has been documented. However, single treatment strategies have not been compared to a placebo group in most of studies, and we do not know the long-term consequences of eosinophilic inflammation, esophageal fibrous remodeling or its possible modifications using different therapies. Furthermore, we lack of a common accepted therapeutic end-point to assess the efficacy of the treatment: from mere resolution of symptoms to full control of esophageal inflammation. This article summarizes the current knowledge about the epidemiology, origin and pathogenesis of the disease, and discuses several practical questions, especially those related to how the affected patients should be treated.

## Introduction

Eosinophilic esophagitis (EoE) is an inflammatory clinicopathological disease first described more than 25 years ago, which has been increasingly recognized over the last decade. More than a half of the current literature on EoE has been published in the last 5 years, and EoE is today recognized as the most common Eosinophilic Gastrointestinal Disease (EGID). EoE must be considered as a common cause into the differential diagnosis of patients manifesting swallowing disorders or esophageal symptoms, because dysphagia and frequent food impaction are the symptoms which usually lead to diagnosis. Esophageal symptoms in EoE seem to be caused by an inflammatory response and not anatomic obstruction [1, 2]. The characteristic histological finding in EoE is large numbers of intraepithelial eosinophils in esophageal biopsies, with a density much higher than in certain patients with gastroesophageal reflux disease (GERD) [3]; however, although both entities can coexist in the same patient, in EoE the symptoms and pathologic features are usually unresponsive to acid suppression treatment. Furthermore, atopic manifestations have been frequently associated to EoE, while were rarely linked to GERD [1, 2].

Current accepted diagnostic criteria for EoE include esophageal and/or upper gastrointestinal tract symptoms accompanied by ≥15 intraepithelial eosinophils/HPF in 1 or more biopsy specimens without pathologic GERD, as shown by normal pH monitoring of the distal esophagus or the lack of response to high-dose proton pump inhibitor (PPI) medication [4].

## Epidemiology

EoE was considered until few year ago to be a rare disease, particularly identified in atopic patients. However, EoE has recently caught the attention of gastroenterologists following its wide recognition in all developed countries. We could say that over the past few decades we have witnessed a sharp rise in the prevalence of EoE as evidenced by the rapid growth in reported cases of EoE in numerous populations. Over the past 10 years there has been an 18-fold rise in the prevalence of EoE in Australia [5] and a 35-fold rise in Philadelphia [6].

The hygiene hypothesis [7] provides a general explanation for the increase in allergic diseases and EGIDs in general, and EoE in particular, parallel to a decrease in infectious diseases. Overly hygienic environments (from controlling exposure to microorganisms during childhood) have led to changes in the patterns of gut microflora and a decrease in exposure to helminthes, causing an imbalance of the immune system and a tendency to develop allergic and autoimmune disorders [8].

A population-based study conducted in Sweden estimated that esophageal eosinophilia were present in about 1% of the adult population [9]. EoE cases reported in the literature are mainly from countries in Europe and North America, and to a lesser extent in Asia, South America, and Australia. No cases have been reported from Africa. This distribution affecting most developed areas parallels bronchial asthma and other atopic conditions; hence we may involve environmental and immune factors in common with other allergy forms in its etiopathogenesis [10]. Beyond of its rising epidemiology, the recognition of the disease by clinicians and pathologists allows more cases correctly diagnosed of EoE, which would also contribute to its rising prevalence.

More than 65% of EoE cases appear during childhood [5], but the condition has also been described in patients of all ages [6]. In contrast to other immunoallergic diseases, EoE predominates in males regardless of age (more than 75% of cases), and most commonly presents in adults during the third to fifth decades of life [7].

## Etiology

Allergy, genes and GERD constitute a combination of possible factors involved in the origin of EoE. EoE has been considered an atopy-associated disorder since its initial descriptions: the majority of patients have evidence of personal or familiar history of asthma, allergic rhinitis, atopic dermatitis, hypersensitivity to foods or aeroallergens, blood eosinophilia or elevated levels of seric IgE [11, 12]. Nowadays there is no doubt about the allergic and chronic nature of EoE, with an inflammation pattern and a profile of cytokine secretion similar to those found in allergic diseases of the respiratory ways [13] and the skin [14], which respond satisfactorily to treatments effective in asthma [15].

In addition to this allergic origin, some recent researches have suggested that gastro-esophageal reflux (GER) could play any etiological role in EoE, by inducing abnormal immunological responses [16]. The real role of GERD in the complex pathophysiology of EoE has not been completely unveil, but further research should define whether these two disorders are independent from each other [17], whether GERD-induced damage may cause EoE, or whether EoE may be determinant for GERD, mainly through motor dysfunction at the distal esophagus or lower esophageal sphincter leading to an impaired clearance of acid. It has been also proposed that acid-suppressive medication could lead to the development of EoE, by facilitating the uptake of underdegraded peptide allergens and increasing gastrointestinal mucosa permeability [18].

Finally, several genes have been involved in EoE specifically those codifying for eotaxin-3 and transforming growth factor TGF-β. Eotaxin-3 was the single gene with the greatest overexpression, but other 574 genes were dysregulated in EoE patients compared to normal individuals in a EoE transcriptome analysis [19]. A single nucleotide polymorphism (SNP) in eotaxin-3 gene has been associated with disease susceptibility [19], another SNP in the promoter of the TGF-β1 gene has been linked to reduced esophageal remodeling following topical steroid treatment. Furthermore, familiar cases of EoE has been commonly reported, and consequently, we can recognize a moderate genetic component in EoE [20] which should be unveil through further research.

In this respect, EoE could either have a multifactorial cause determined by the exposure of the digestive mucosa of the immune system to food or airborne allergens, modulated in certain cases by the exposure of genetically predisposed individuals to acid. The contribution of these possible etiological factors to the development of these various diseases is crucial to define specific treatments.

## Pathophysiology

Pathogenic mechanisms of EoE has been related with atopy, because a Th2-type immunologic response has been demonstrated in EoE, in which T cells, interleukin (IL)-5 expression, eosinophils and positive IgE immunostaining were shown to characterize the inflammatory infiltrate [21], in a similar way it appears in bronchial asthma. Th2-type responses are mediated by T helper CD4 cell and driven by cytokines, such as IL-4, -5, 9 and IL-13, whose potential role in EoE has been supported in a number of basic studies [22].

Experimental murine models of EoE have shown that allergen exposition leads to EoE following molecular mechanisms involving Th2-type specific responses. IL-5 seems to be critical for the development of the disease, because transgenic mice that overexpress IL-5 showed an increase in circulating blood eosinophils and an intense accumulation in the esophageal *lamina propria*, which was proportional to the serum concentration of IL-5 [23, 24] when stimulated with inhaled [25, 26] of epicutaneous allergens [27]. By contrast, IL-5 deficient mice did not develop EoE when exposed to airborne allergens [26]. The percentage of blood-circulating IL-5^+^CD4 T cells in humans correlated with the severity of esophageal eosinophilia [28].

IL-13 is a immunoregulatory cytokine involved in several allergic diseases, whose role has been also studied in EoE: A 16-fold increase in IL-13 mRNA expression has been observed in EoE patients compared to healthy individuals; in human esophageal epithelial cell cultures IL-13 are capable of partially reproduce the characteristic EoE transcripsome, and IL-13 enhance the gene expression of the eosinophil-activating chemoattractants eotaxin-1 and 3 [29]. In mice, intratracheal delivery of IL-13 induced esophageal eosinophilia in a dose-dependent manner [30].

Th2-type cytokines are powerful activators of the production of antibodies by B cells, specially IgE, through the stimulation of IL-4 and IL-13 [31], a process that can be particularly important in the pathophysiology of EoE, we have evidence for *in situ* IgE production and class switching to IgE in the esophageal mucosa of EoE patients [32], which appears to be present into the esophageal epithelium linked to the surface of activated mast cells [33-37].

Eosinophils are functionally complex cells, which possess both regulator and effector functions, these last are exerted by means of the preformed cytotoxic proteins stored in their cytoplasmatic granules (Major Basic Protein, Eosinophil peroxidase, Eosinophil derived neurotoxin, Eosinophil Cationic protein) and lipid mediators (platelet-activating factor, leukotriene C_4_) that induce the activation of vascular endothelium and contribute to cellular dysfunction [24]. The cytotoxic role of eosinophils in EoE is directly related with the observed histopathological changes in the mucosa of the organ [38, 39], with destruction of the most superficial epithelial layers (in contact with the lumen of the esophagus) and the regenerative response from the basal layers of the epithelium. At the same time, eosinophils themselves can contribute to esophageal motor disturbances which clinically characterize EoE, through the action of MBP as a powerful agonist of the M2 receptors of acetylcholine that govern the function of the smooth esophageal muscle [40, 41]. In asthma, eosinophils are implicated in the remodeling of the bronchial wall through the release of toxic mediators from its cytoplasmic granules [42]. Similarly, fibrous esophageal remodeling has been described in children with EoE, in which subepithelial collagen is deposited through a mechanism dependent of TGF-β [43, 44].

## Symptoms

EoE is characterized by a spectrum of presenting symptoms. An extensive review of EoE reported in 2002 [45] found that symptoms in adults included dysphagia, food impaction, vomiting, and chest pain, whereas children also have nausea, heartburn, epigastric pain, sialorrhea, food aversion, delayed growth, and respiratory complaints (cough, stridor, sinusitis, obstruction, pneumonia). Patients commonly have a number of simultaneous EoE-related symptoms at any age.

Differences in symptoms according to patient age could be explained as different functional phenotypes determined by eosinophilic esophageal inflammation, but the existence of a time sequence for EoE in which symptoms are developed chronologically should be also considered as a more plausible option [46]: In children, the ability to effectively report symptoms determines various presentation forms [47], thus, smaller children (who cannot report dysphagia) would have a number of eating disorders including food aversion or failure to thieve; later on, vomiting, regurgitation, and both chest and abdominal pain, mimicking GERD; from 11 years on, the condition would manifest with dysphagia and food impaction, which predominate in adults. In adult patients intermittent dysphagia is the most common complaint, and occurs in more than 70% of cases in some series; however, food impaction is the symptom that most often leads to a diagnosis (56 to 88% of cases) [48]. While less frequent, GERD symptoms are also commonplace [49]. Overall, symptoms persist for a long time, even years, before a diagnosis is reached [12, 50].

It should be noted that EoE patients eat dead slow, taking much longer that the rest of the family to complete a meal, holding food in the mouth, and usually drink after each and every bite; especially in the case of the specific more problematic foods such as meat or bread [47]; parents should be asked for this during history-taking.

## Endoscopy

EoE has been, and still is, an underdiagnosed condition in many settings, since endoscopic findings are usually much subtler than those seen in esophageal growths or erosive disorders [37]. A careful exam is therefore needed that should include biopsy samples from all suspect cases in order to ensure a proper diagnosis [38]. From an endoscopic viewpoint, EoE has a great variety of potential findings [39-41]. Literature reports include reduced esophageal caliber [42] as focal or segmentary stenoses, trachealized esophagus, irregular mucosa, reddish mucosa, whitish elevated papules that resemble candidiasis [43], longitudinal linear furrows (also called esophageal corrugation) [44], changes in esophageal mucosal pattern [45], mucosal frailty [46], esophageal tears [47], and food impaction [11, 30, 36]. A retrospective review of 117 patients with a histological diagnosis of EoE showed that the esophagus had been reported normal in up to 24.79% of cases [39], and a prospective analysis of adult patients presenting with dysphagia showed that 42% of patients with final diagnosis of EoE had normal looking esophagus [51]. This data clearly suggest that changes in this organ’s appearance may be subtle enough to be overlooked by an endoscopist not used to this disease. This highly variable range changes seen on the organ’s surface translate the different severities of histological epithelial lesions, and a direct correlation between endoscopic severity, histological severity, and eosinophilic inflammatory infiltration density and activation has been reported [34].

**Figure 1:**
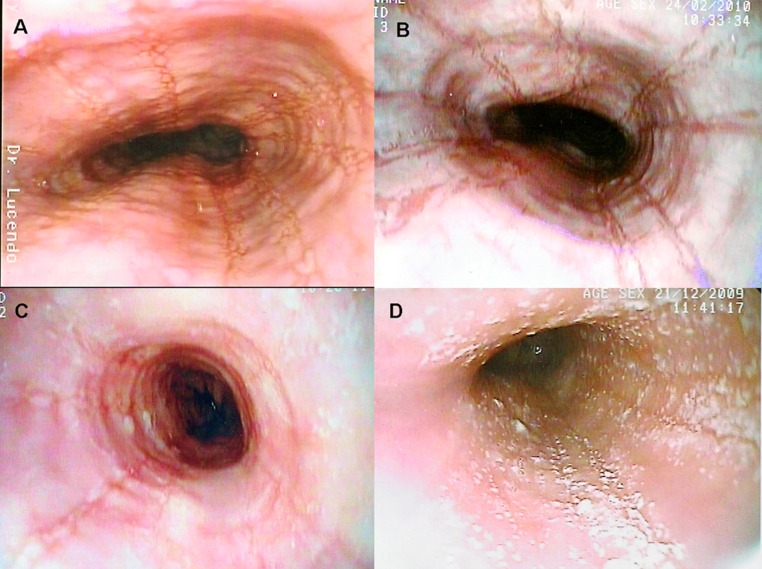
Several endoscopic aspects of eosinophilic esophagitis. A: Normal-caliber esophagus with longitudinal linear furrows and irregular mucosa. B: Fragile-looking mucosa, which comes loose with biopsy forceps, and marked mucosal corrugation. C: Reduced-caliber, trachealized esophagus with irregular, cobblestone appearance. D: The esophageal mucosal surface may be covered in cotton-like exudates mimicking candiadiasis, but biopsy finds them to be multiple eosinophil-containing micro-abscesses.

## Histology

The presence of eosinophils in the esophageal epithelium may be seen in many esophageal conditions [48], and of itself defines no particular disease, but should be assessed within the patient’s clinical and pathological context. Eosinophilic infiltration in EoE involves the entire esophagus, but often in a patchy manner. It is for this reason that multiple biopsies at different level are required for an accurate diagnosis. Various papers have reported that the density of eosinophilic infiltration is similar in the distal and proximal thirds of the esophagus [49, 50], and a good diagnostic strategy involves collecting samples from both these thirds [1]. Number of biopsies is relevant for diagnostic sensitivity, as the latter increases with sample number and reaches 100% with 5 biopsy specimens [51].

The most characteristic finding is a high number of eosinophils infiltrating the esophageal epithelium. The usual assessment approach is their count in fields more densely inflamed using an x400 lens (number per highpower field, HPF, x400), a non-standized measure as the area included in a HPF varies from one microscope manufacturer to the next. The threshold number of eosinophils in diagnosing EoE also varies among authors [6, 11, 52-55], but it is currently accepted that 15 eosinophils/HPF would suffice in the presence of a consistent clinical context when other histopathological findings are noted [1]. Eosinophils may be diffusely distributed throughout the epithelial thickness, but tend to be more numerous in apical strata near the esophageal lumen [50]. In cases with higher numbers they usually coalesce and make up micro-abscesses [56], which may eventually destroy the superficial epithelium [34].

Extracellular eosinophilic granules and major basic protein (MBP) deposition, both extracellularly [57] and within the cytoplasm [30, 50, 58], may be seen. Micro-abscesses, extracellular deposition of eosinophilic proteins, and positive immunostaining for MBP are findings exclusive of EoE that are not seen in GERD [59]. Good biopsies allow the study of other histopathological findings characteristic of EoE, including basal layer hyperplasia with acanthosis or presence of proliferative stratum cells in higher epithelial levels, elongated papillas in the *lamina propria*, and intercellular edema, reflected by enlarged intercellular spaces. These findings translate a nonspecific, proliferative epithelial response [34], as may also be seen in GERD [60-62]. Subepithelial collagen deposition has been reported within the esophageal *lamina propria* of pediatric patients with EoE to a significantly greater extent *versus* normal conditions and GERD [57, 63]. Epithelial histopathological findings usually regress to normal after therapy.

**Figure 2:**
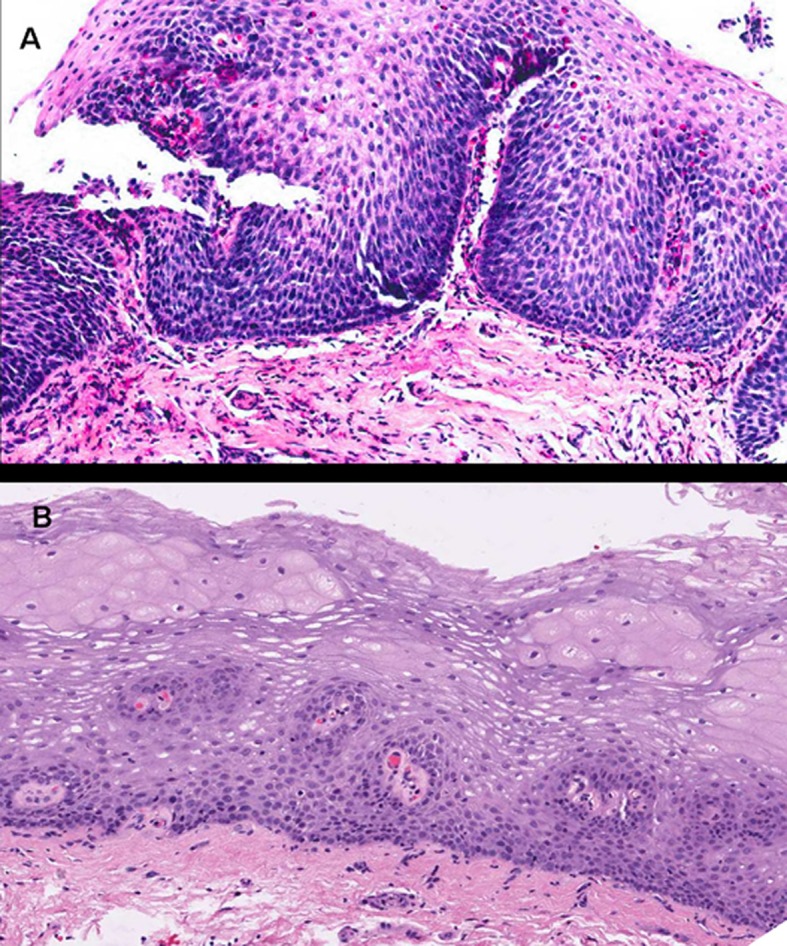
Images corresponding to the same patient before and after therapy with topical fluticasone propionate to illustrate changes in the esophageal epithelium. A: Marked epithelial proliferation with basal-cell hyperplasia reaching up to superficial strata, in addition to elongated connective papillas, which appear thicker and hypervascularized. Numerous eosinophils infiltrate into the epithelium. B: Obtained after 3 months under treatment with fluticasone propionate. The esophageal epithelium exhibits fewer cells and recovered stratification, with basal cells occupying not more than 15% of esophageal thickness, and no eosinophilic infiltration (hematoxylin and eosin, x200).

## Treatment

Although EoE has gained a great importance in recent years, we currently lack commonly accepted treatment strategies and the adequate management of patients has been somewhat controversial. In addition, no randomized controlled studies are available, except for two studies of pediatric patients [52, 53] and a recent study on adults [54], and it is difficult to control all the etiological factors which could contribute to the development of EoE. Furthermore, there is scant knowledge of the long-term effects of the different therapies to control the inflammation of the organ and of their ability to modify the natural history of the disease.

The therapies tested include: a) those focused on eliminating potential triggering allergens from the diet which, although might be useful, have disadvantages; b) various drugs beneficial for other inflammatory conditions but which have not been officially indicated for EoE; and c) endoscopic treatment which, through esophageal dilation, aims to correct the alterations of the caliber of the esophagus.

### Dietary management and control of antigenic exposure

Studies initially performed on children with EoE showed that allergies to certain foods contributed significantly to its pathogenesis and that symptoms and histopathological findings improved in most cases once certain foods had been eliminated. Elemental diet (based on amino acids and lacking of antigenic capacity) was first used with a group of children with EoE attributed to GER in 1995 and led to total clinical and histological remission in 80% of cases (and partial in the rest) over a 6-week treatment period and the symptoms reappeared when they resumed their normal diets [55]. These results were after corroborated in other several studies [6, 56], establishing that childhood EoE could be considered as a food allergy. Aside from being the gold standard in the treatment of EoE, the elemental diet has a number of disadvantages such as it is expensive, has an unpleasant taste, which in many cases means that it must be fed through a nasogastric tube in up to 80% of patients in the most recent study [6], and it cannot be administered for adults and in chronic uses.

Directed elimination diets consisted in identifying and exclusively excluding those foods which triggered EoE. Allergic food cannot be identified through clinical history because the patient does not usually associate certain foods with the development of symptoms, and specific allergy tests should be developed, by detecting specific IgE levels in the blood, or by skin prick tests (SPT) and/or atopic patch tests (APT). This strategy can sometimes be complicated, given that the physiopathology of EoE seems to be mediated by a delayed hypersensitivity reaction and food allergies in a patient are not necessarily responsible for eosinophilic inflammation of the organ. In 2002 Spergel *et al* used SPTs and APTs on patients with EoE for the first time to control the elimination diet and obtained positive results especially with regard to the following food allergens in each test, in SPTs: milk, eggs, peanuts, seafood, peas, beef, fish, rye, wheat and tomatoes; in APTs: wheat, corn, beef, milk, soy, rye, eggs, chicken, oats and potatoes [57]. This study group subsequently eliminated the foods which triggered an allergic reaction [58]. Of the 146 EoE pediatric patients studied, specific foods were identified in 77 cases by the allergy tests and after they were eliminated, 77% managed to control the disease while 10% showed no improvement. Unfortunately, we have no information on similar studies in adults.

However, most patients need to exclude more than one food type from the diet, which can lead to significant nutritional deficiency that would have to be substituted appropriately, especially in children. Food reintroduction is a very important part of the dietary management of EoE patients, which should be always considered after normal esophageal biopsies have been obtained in patients following elemental or elimination diets. Food reintroduction aims to improve patients’ acceptance of a less restrictive diet and selectively identify causative foods for EoE. For the latter reason, a reintroduction sequence must be planned, beginning with those unlikely to cause EoE foods, such as vegetables and fruit, and following with those which are most likely to cause EoE, such as corn, chicken, wheat, beef, milk, soy or eggs [59]. Endoscopic exams and biopsies should be carried out every 1 - 2 months to assess the absence of inflammation, or as soon as the patient develops esophageal symptoms.

Exclusion diets, the third dietary treatment strategy, was developed with the intention of avoiding allergic tests, and consist in eliminating the foods most likely to cause allergies, i.e. those are most allergenic, regardless of the individual allergy test results. In 2006, 6 foods (cows’ milk protein, soy, wheat, eggs, peanuts and seafood) were excluded from a cohort of 35 pediatric patients diagnosed with EoE, who were compared to another group of 24 EoE patients following an elemental diet, both over a 6-week period [60]. Clinical improvement was observed and there was a decrease in the esophageal infiltrate by eosinophils in 74% of the patients under exclusion diets and in 88% of the patients on elemental diets. These favorable results were not subsequently achieved in a small study carried out on adults using the same strategy in which clinical improvement was only evidenced by a symptoms score decrease of 30% with incomplete histological resolution [61]. In conclusion, the empirical exclusion of the aforementioned 6 foods from the diet is an efficient treatment method well-tolerated in children with EoE as it allows solid food to be consumed but cannot be recommended as a sole treatment for adults.

### Pharmacological treatment in EoE

At present, since no drugs have been specifically approved for use in EoE, we must resort to medication used for other allergic diseases. EoE is also a chronic disease that can require long-term treatment, which is why therapies must be assessed both in terms of efficiency and safety to avoid or minimize their potential side effects. Furthermore, none of the therapies currently available have modified the course of the disease or totally cured its symptoms in the long-term. Despite these problems, the need to provide treatment for patients with EoE has led to the use of different drugs in recent years, as follows:

1) Proton pump inhibitors (PPIs): These are not considered to be a specific treatment for EoE but are useful to distinguish EoE from GORD [4, 17, 62] and can also offer clear benefits for some patients diagnosed with EoE with secondary symptoms of GERD, most likely resulting from poor esophageal acid clearance [63] caused by motor alterations associated with eosinophilic inflammation of the organ. However, 2 recent studies have shown that PPI-based therapies could be effective in the short-term in some patients. In the first, 3 patients with EoE became asymptomatic and normalized endoscopic and histological finding after being treated with PPIs for 2 months [64]. The second study prospectively randomized 30 adults patients to be treated with swallowed fluticasone or with esomeprazole over 8 weeks, it was unable to show differences between both treatments, both leading to a decrease in the number of eosinophils and an improvement of approximately 50% in dysphagia and partial histological resolution [54]. However, as the study was small, this could have limited its ability to detect a significant difference. In view of these results, PPIs can be recommended as a co-therapy for some patients and not only to differentiate EoE from GERD. Nevertheless, we do not know what effect gastric acid antisecretory treatment could have on the symptoms and histopathological findings of EoE over the medium and long term and are unable to rule out that the symptoms could re-appear after some time, if the food or airborne antigenic exposure determining the disease were to prevail.

2) Systemic Corticoids: Various previous studies have shown the efficiency of systemic corticoids in controlling the symptoms and esophageal inflammatory infiltrate in EoE, but since this is a chronic illness, the use of systemic steroids is not recommended due to their adverse effects giving preference to other safer therapies. Oral prednisone and different dosages of methylprednisolone [53] were highly effective, although the symptoms and the esophageal eosinophilic infiltrate reappeared several months after the treatment was discontinued. A recent study compared oral prednisone to a topical fluticasone propionate, demonstrating equally effectiveness in both cases but higher side effects in the group treated with prednosine [53]. Therefore, systemic corticosteroids would only be recommended in severe, refractory or urgent cases of EoE.

3) Topical steroids: This is the front-line treatment for many EoE cases [65, 66] and fluticasone propionate is the most widely used. Since it was first used in EoE [67], numerous studies [15, 33, 34, 39, 40, 48, 52, 53, 68-72] have shown that it is efficient in children and adults but has minimum side effects, the most common being pharyngeal-esophageal candidiasis.

The only one available randomized, double-blind, placebo-controlled trial using fluticasone propionate in pediatric patients with EoE showed that 50% of patients treated with fluticasone (880 µg divided twice daily during 3 months) had histological remission, a significant decrease in the densities of eosinophils and CD8^+^ lymphocytes, especially in the proximal third of the esophagus [52].

The dosages of fluticasone propionate used in the different articles published on EoE range from 176 µg/day in children to 1 g/day in adults (two dosages given), over a period of 6 -12 weeks. The main disadvantage of this treatment is that it is difficult to administer (normally by inhaler, it must be applied on the tongue and then swallowed to treat EoE). Consequently, it is very important to teach patients how to take the drug correctly and inform them that they should not eat or drink for at least 30 minutes afterwards. We have a liquid form of fluticasone, initially intended for nasal inhalation, which makes it easier to swallow. A viscous budesonide solution was used to make it easier to administer correctly, particularly for children, achieving positive results in 80% of the patients; no safety issues relating to the drug were reported [71]. The budesonide dosages used for these pediatric patients were 1 - 2 mg/day in a volume of 8-12 ml, taken once per day.

4) Other antiallergic therapies: Disodium cromoglycate did no show clinical nor histological improvement in a study on 14 children with EoE who were given dosages of 100 mg/day over 1 month [6], and, accordingly, we do not have enough evidence to recommend that this treatment be used in EoE. Montelukast, a leukotriene receptor antagonist used to treat bronchial asthma was used in a small group of 8 patients with EE who were given high dosages (up to 100 mg/day) [73]. After several weeks of treatment, 7 patients admitted remission of symptoms in a telephone-based questionnaire but none showed significant histological improvement. Probably this therapy could not be useful because no differences in gene expression levels of the cysteinyl leukotrienes in the esophageal epithelium were found between children with EoE and normal controls [74]. It seems clear that Montelukast does not lead to histological remission of EoE, although further studies are required to determine the potential efficiency of maintaining steroid-induced remission.

5) Azathioprine/6-mercaptopurine: The only one published study was performed over 3 steroid-dependent adults with EoE, who were treated with AZA or 6-MP (2 - 2.5 mg/kg/day) in a similar way to in inflammatory bowel disease, showing remission of symptoms and of the eosinophilic infiltrate which continued during the course of the therapy (3 - 8 years) without the need for steroids [75]. After the treatment was discontinued, the disease recurred in two patients. Further research based on a larger number of cases is required to evaluate the effectiveness of the treatment.

6) Experimental therapies and future therapies: Knowledge of the molecular mechanisms of the development of EoE has paved the way for the use of monoclonal antibodies against cytokines which mediate the physiopathology of the disease. Some experimental work has been carried out which assesses the use of these possible therapies. The most promised therapy was MepolizumAb, a humanized monoclonal antibody against interleukin-5 (IL-5) which had been successfully used in the treatment of hypereosinophilic syndrome [76, 77]. In a recently conducted double-blind, randomized, placebo-controlled clinical trial on adult patients with EoE, those treated with MepolizumAb showed a significant reduction in mean esophageal eosinophilia (-54%) compared to the placebo group (-5%) four weeks after beginning treatment but there was no further decrease after additional doses. The expression of molecules associated with esophageal remodeling (TGF-β and tenascin C) was reversed, but these changes led to minimal symptomatic improvement in EoE patients [78]. Other biological therapies, as OmalizumAb (a monoclonal anti-IgE antibody) [79, 80] and InfliximAb (a chimeric monoclonal anti tumor necrosis factor -α antibody) [81] were not effective in treating EoE in any of the few studies performed.

### Endoscopic treatments for EoE

EoE has been associated to extremely fragile mucosa, because a high rate of tears and lacerations has been described, as a complication of endoscopic procedures as well as a result of patients’ attempts to induce vomiting and dislodge impacted food. Cases of spontaneous esophageal perforation [82] and Boerhaave’s syndrome [83] have even been reported, after the mere passage of the endoscope [84] in patients with EoE, so the various endoscopic procedures should be gently performed.

Food impaction is the clinical manifestation which most frequently leads to diagnosis of EoE in adult patients [48]. An analysis of 251 Swiss patients with EoE showed that 34.7% required extraction of the impacted bolus using flexible or rigid esophagoscopy, observing a 20% rate of transmural perforations [85] using the latter and, therefore, bolus removal by rigid endoscopy is a high-risk procedure and should be avoided in EoE patients.

Narrowing of the esophageal lumen is frequently observed in EoE, because of that mechanical dilation using hydropneumatic dilators or bougies has been carried out by various authors as a treatment option for the disease in a similar way to how it is used in other cases of rigid or fibrous stenosis like in GERD or following caustication. A study of literature shows that esophageal dilation is an efficient treatment, providing immediate symptomatic relief [86, 87]. However, these procedures have been warned to pose a higher risk of complications in patients with EoE. The long evolution of dysphagia, esophageal stenosis and the high density of eosinophils have been suggested to be predictive factors of these complications during dilation [88]. Most cases of esophageal perforation (spontaneous or after endoscopic procedures) described only led to pneumomediastinum [89, 90], but in two cases, an emergency esophagectomy via thoracotomy and esophagogastroplasty were required, in one case after esophageal bouginage [91] and in another following spontaneous rupture [92]. No patient fatalities have been reported, but in order to minimize complications, it seems practical to proceed slowly and carefully and dilate using smaller calibers than those used in different types of stenosis.

On the other hand, endoscopic dilation is a mechanical procedure which has no effect on the underlying inflammatory process [93], and, accordingly, its efficiency can be limited over time. The duration of the effect in published cases cannot be appropriately estimated due to the short monitoring period, although it usually ranges from 3 - 12 months and it is very normal for patients to have to undergo repeated dilation (up to 5 times ) to control their symptoms [93, 94].

Consequently, endoscopic dilation can be a risky technique in these patients [93], and should be considered as an alternative treatment for patients with EoE and esophageal stenosis when other measures have failed, especially topical steroids [95].

## Practical considerations

Drugs, diets and endoscopic dilations seem to be able to relive symptoms of EoE, but the information at our disposal on the efficacy of the different treatments is based on a limited number of patients monitored over short periods of time. The single treatment strategies have not been compared to a placebo group in most of studies and most available information is related to pediatric EoE, the results of which are subsequently extrapolated to adults. We do not know the long-term consequences of eosinophilic inflammation, fibrous remodeling of the esophagus or possible modifications using different therapies. What is more, we lack of a common accepted therapeutic end-point to assess the efficacy of the treatment, from mere resolution of symptoms to full control of esophageal inflammation.

For these reasons, it is difficult to recommend common guidelines for all patients. The experience of each centre and the availability of techniques and studies also limit the treatment options as well as the objectives established or achievable in each case. Guidelines elaborated by EoE experts recommended to treat asymptomatic cases of EoE in order to prevent the potential consequences of fibrous remodeling of the organ [4], although its consequences over the long-term are not known. In any event, in the absence of treatment, we should consider EoE as a chronic disease with intermittent symptoms and histological inflammation which persists over time and has repercussions on the patients’ quality of life [96].

Due to the co-existence of GER in many cases of EoE and the effect of acid-secretion inhibitors when controlling the symptoms, in the event of suspected EoE, it would be reasonable to carry out a therapeutic test using PPIs for a period of 8 weeks before repeating the endoscopy and taking further biopsies. We will only be able to propose a specific treatment when the persistence of the eosinophilic inflammatory infiltrate and the symptoms deriving therefrom have been verified [97].

Swallowed topical steroids could be the number one alternative, both in children and adults with EoE, while carrying out the relevant sensitivity studies to allergens. The use of liquid formulas (liquid fluticasone is available for intranasal administration) or viscous compounded medication minimizes the difficulty in swallowing these drugs, especially in children.

Treatment with dietary changes should always be considered, with the aid of a nutritionist, especially in children, guaranteeing a balanced diet to avoid nutritional deficiency. In adults, the elemental diet is not a real alternative and the few experiences involving food exclusion have not been widely studied, although perhaps it could be less effective due to the increasing participation of airborne allergens in the physiopathology of EoE in adults [61]. At centers where in-depth food sensitivity studies can be performed, empirical elimination diets should be tested using the foods causing sensitivity. It should be noted that these studies are not very standardized.

As mentioned previously, endoscopic dilation would only be considered in cases of persistent symptoms and a reduction in the caliber of the esophagus which have failed to respond to previous therapies and should be performed gently using medium-sized bougies.

The various therapeutic approaches to EoE suggest that none have absolute advantages. Options should therefore be chosen on a case-by-case basis once the patients’ characteristics, parents and patient’s preferences and sensitivity to allergens are known.

## References

[1] Atkins D, Kramer R, Capocelli K, Lovell M, Furuta GT (2009). Eosinophilic esophagitis: the newest esophageal inflammatory disease. Nat Rev Gastroenterol Hepatol.

[2] Straumann A, Spichtin HP, Bernoulli R, Loosli J, Vogtlin J (1994). [Idiopathic eosinophilic esophagitis: a frequently overlooked disease with typical clinical aspects and discrete endoscopic findings]. Schweiz Med Wochenschr.

[3] Winter HS, Madara JL, Stafford RJ, Grand RJ, Quinlan JE, Goldman H (1982). Intraepithelial eosinophils: a new diagnostic criterion for reflux esophagitis. Gastroenterology.

[4] Furuta GT, Liacouras CA, Collins MH, Gupta SK, Justinich C, Putnam PE, Bonis P (2007). Eosinophilic esophagitis in children and adults: a systematic review and consensus recommendations for diagnosis and treatment. Gastroenterology.

[5] Cherian S, Smith NM, Forbes DA (2006). Rapidly increasing prevalence of eosinophilic oesophagitis in Western Australia. Arch Dis Child.

[6] Liacouras CA, Spergel JM, Ruchelli E, Verma R, Mascarenhas M, Semeao E, Flick J (2005). Eosinophilic esophagitis: a 10-year experience in 381 children. Clin Gastroenterol Hepatol.

[7] Garn H, Renz H (2007). Epidemiological and immunological evidence for the hygiene hypothesis. Immunobiology.

[8] Weinstock JV, Elliott DE (2009). Helminths and the IBD hygiene hypothesis. Inflamm Bowel Dis.

[9] Ronkainen J, Talley NJ, Aro P, Storskrubb T, Johansson SE, Lind T, Bolling-Sternevald E (2007). Prevalence of oesophageal eosinophils and eosinophilic oesophagitis in adults: the population-based Kalixanda study. Gut.

[10] Furuta GT, Forbes D, Boey C, Dupont C, Putnam P, Roy S, Sabra A (2008). Eosinophilic gastrointestinal diseases (EGIDs). J Pediatr Gastroenterol Nutr.

[11] Simon D, Marti H, Heer P, Simon HU, Braathen LR, Straumann A (2005). Eosinophilic esophagitis is frequently associated with IgE-mediated allergic airway diseases. J Allergy Clin Immunol.

[12] Sgouros SN, Bergele C, Mantides A (2006). Eosinophilic esophagitis in adults: a systematic review. Eur J Gastroenterol Hepatol.

[13] Bousquet J, Vignola AM, Demoly P (2003). Links between rhinitis and asthma. Allergy.

[14] Simon D, Vassina E, Yousefi S, Kozlowski E, Braathen LR, Simon HU (2004). Reduced dermal infiltration of cytokine-expressing inflammatory cells in atopic dermatitis after short-term topical tacrolimus treatment. J Allergy Clin Immunol.

[15] Noel RJ, Putnam PE, Collins MH, Assa'ad AH, Guajardo JR, Jameson SC, Rothenberg ME (2004). Clinical and immunopathologic effects of swallowed fluticasone for eosinophilic esophagitis. Clin Gastroenterol Hepatol.

[16] Spechler SJ, Genta RM, Souza RF (2007). Thoughts on the complex relationship between gastroesophageal reflux disease and eosinophilic esophagitis. Am J Gastroenterol.

[17] Molina-Infante J, Ferrando-Lamana L, Mateos-Rodriguez JM, Perez-Gallardo B, Prieto-Bermejo AB (2008). Overlap of reflux and eosinophilic esophagitis in two patients requiring different therapies: a review of the literature. World J Gastroenterol.

[18] Merwat SN, Spechler SJ (2009). Might the use of acid-suppressive medications predispose to the development of eosinophilic esophagitis?. Am J Gastroenterol.

[19] Blanchard C, Wang N, Stringer KF, Mishra A, Fulkerson PC, Abonia JP, Jameson SC (2006). Eotaxin-3 and a uniquely conserved gene-expression profile in eosinophilic esophagitis. J Clin Invest.

[20] Lucendo AJ, Lucendo B (2010). An update on the immunopathogenesis of eosinophilic esophagitis. Expert Rev Gastroenterol Hepatol.

[21] Straumann A, Bauer M, Fischer B, Blaser K, Simon HU (2001). Idiopathic eosinophilic esophagitis is associated with a T(H)2-type allergic inflammatory response. J Allergy Clin Immunol.

[22] Blanchard C, Rothenberg ME (2008). Basic pathogenesis of eosinophilic esophagitis. Gastrointest Endosc Clin N Am.

[23] Rothenberg ME (2004). Eosinophilic gastrointestinal disorders (EGID). J Allergy Clin Immunol.

[24] Rothenberg ME, Mishra A, Brandt EB, Hogan SP Gastrointestinal eosinophils. Immunol Rev.

[25] Mishra A, Hogan SP, Brandt EB, Rothenberg ME (2001). An etiological role for aeroallergens and eosinophils in experimental esophagitis. J Clin Invest.

[26] Mishra A, Hogan SP, Brandt EB, Rothenberg ME (2002). IL-5 promotes eosinophil trafficking to the esophagus. J Immunol.

[27] Akei HS, Mishra A, Blanchard C, Rothenberg ME (2005). Epicutaneous antigen exposure primes for experimental eosinophilic esophagitis in mice. Gastroenterology.

[28] Bullock JZ, Villanueva JM, Blanchard C, Filipovich AH, Putnam PE, Collins MH, Risma KA (2007). Interplay of adaptive th2 immunity with eotaxin-3/c-C chemokine receptor 3 in eosinophilic esophagitis. J Pediatr Gastroenterol Nutr.

[29] Neilsen CV, Bryce PJ Interleukin-13 directly promotes oesophagus production of CCL11 and CCL24 and the migration of eosinophils. Clin Exp Allergy.

[30] Mishra A, Rothenberg ME (2003). Intratracheal IL-13 induces eosinophilic esophagitis by an IL-5, eotaxin-1, and STAT6-dependent mechanism. Gastroenterology.

[31] Barner M, Mohrs M, Brombacher F, Kopf M (1998). Differences between IL-4R alpha-deficient and IL-4-deficient mice reveal a role for IL-13 in the regulation of Th2 responses. Curr Biol.

[32] Vicario M, Blanchard C, Stringer KF, Collins MH, Mingler MK, Ahrens A, Putnam PE Local B cells and IgE production in the oesophageal mucosa in eosinophilic oesophagitis. Gut.

[33] Lucendo AJ, Navarro M, Comas C, Pascual JM, Burgos E, Santamaria L, Larrauri J (2007). Immunophenotypic characterization and quantification of the epithelial inflammatory infiltrate in eosinophilic esophagitis through stereology: an analysis of the cellular mechanisms of the disease and the immunologic capacity of the esophagus. Am J Surg Pathol.

[34] Teitelbaum JE, Fox VL, Twarog FJ, Nurko S, Antonioli D, Gleich G, Badizadegan K (2002). Eosinophilic esophagitis in children: immunopathological analysis and response to fluticasone propionate. Gastroenterology.

[35] Lucendo AJ, Bellon T, Lucendo B (2009). The role of mast cells in eosinophilic esophagitis. Pediatr Allergy Immunol.

[36] Justinich C, Katz A, Gurbindo C, Lepage G, Chad Z, Bouthillier L, Seidman E (1996). Elemental diet improves steroid-dependent eosinophilic gastroenteritis and reverses growth failure. J Pediatr Gastroenterol Nutr.

[37] Kirsch R, Bokhary R, Marcon MA, Cutz E (2007). Activated mucosal mast cells differentiate eosinophilic (allergic) esophagitis from gastroesophageal reflux disease. J Pediatr Gastroenterol Nutr.

[38] Dahms BB (2004). Reflux esophagitis: sequelae and differential diagnosis in infants and children including eosinophilic esophagitis. Pediatr Dev Pathol.

[39] Lucendo Villarin AJ, Carrion Alonso G, Navarro Sanchez M, Martin Chavarri S, Gomez Senent S, Castillo Grau P, Pascual Turrion JM (2005). Eosinophilic esophagitis in adults, an emerging cause of dysphagia. Description of 9 cases. Rev Esp Enferm Dig.

[40] Lucendo AJ, Castillo P, Martin-Chavarri S, Carrion G, Pajares R, Pascual JM, Mancenido N (2007). Manometric findings in adult eosinophilic oesophagitis: a study of 12 cases. Eur J Gastroenterol Hepatol.

[41] Lucendo AJ (2006). Motor disturbances participate in the pathogenesis of eosinophilic oesophagitis, beyond the fibrous remodelling of the oesophagus. Aliment Pharmacol Ther.

[42] Gleich GJ, Kita H (1997). Bronchial asthma: lessons from murine models. Proc Natl Acad Sci U S A.

[43] Aceves SS, Newbury RO, Dohil R, Bastian JF, Broide DH (2007). Esophageal remodeling in pediatric eosinophilic esophagitis. J Allergy Clin Immunol.

[44] Chehade M, Sampson HA, Morotti RA, Magid MS (2007). Esophageal subepithelial fibrosis in children with eosinophilic esophagitis. J Pediatr Gastroenterol Nutr.

[45] Fox VL, Nurko S, Furuta GT (2002). Eosinophilic esophagitis: it's not just kid's stuff. Gastrointest Endosc.

[46] Noel RJ, Putnam PE, Rothenberg ME (2004). Eosinophilic esophagitis. N Engl J Med.

[47] Putnam PE (2008). Eosinophilic esophagitis in children: clinical manifestations. Gastrointest Endosc Clin N Am.

[48] Lucendo AJ, Pascual-Turrion JM, Navarro M, Comas C, Castillo P, Letran A, Caballero MT (2007). Endoscopic, bioptic, and manometric findings in eosinophilic esophagitis before and after steroid therapy: a case series. Endoscopy.

[49] Katzka DA (2008). Demographic data and symptoms of eosinophilic esophagitis in adults. Gastrointest Endosc Clin N Am.

[50] Lucendo Villarin AJ (2009). Eosinophilic esophagitis — clinical manifestations, diagnosis, and treatment. Rev Esp Enferm Dig.

[51] Mackenzie SH, Go M, Chadwick B, Thomas K, Fang J, Kuwada S, Lamphier S (2008). Eosinophilic oesophagitis in patients presenting with dysphagia—a prospective analysis. Aliment Pharmacol Ther.

[52] Konikoff MR, Noel RJ, Blanchard C, Kirby C, Jameson SC, Buckmeier BK, Akers R (2006). A randomized, double-blind, placebo-controlled trial of fluticasone propionate for pediatric eosinophilic esophagitis. Gastroenterology.

[53] Schaefer ET, Fitzgerald JF, Molleston JP, Croffie JM, Pfefferkorn MD, Corkins MR, Lim JD (2008). Comparison of oral prednisone and topical fluticasone in the treatment of eosinophilic esophagitis: a randomized trial in children. Clin Gastroenterol Hepatol.

[54] Peterson KA, Thomas KL, Hilden K, Emerson LL, Wills JC, Fang JC (2009). Comparison of Esomeprazole to Aerosolized, Swallowed Fluticasone for Eosinophilic Esophagitis. Dig Dis Sci.

[55] Kelly KJ, Lazenby AJ, Rowe PC, Yardley JH, Perman JA, Sampson HA (1995). Eosinophilic esophagitis attributed to gastroesophageal reflux: improvement with an amino acid-based formula. Gastroenterology.

[56] Markowitz JE, Spergel JM, Ruchelli E, Liacouras CA (2003). Elemental diet is an effective treatment for eosinophilic esophagitis in children and adolescents. Am J Gastroenterol.

[57] Spergel JM, Beausoleil JL, Mascarenhas M, Liacouras CA (2002). The use of skin prick tests and patch tests to identify causative foods in eosinophilic esophagitis. J Allergy Clin Immunol.

[58] Spergel JM, Andrews T, Brown-Whitehorn TF, Beausoleil JL, Liacouras CA (2005). Treatment of eosinophilic esophagitis with specific food elimination diet directed by a combination of skin prick and patch tests. Ann Allergy Asthma Immunol.

[59] Spergel JM, Shuker M (2008). Nutritional management of eosinophilic esophagitis. Gastrointest Endosc Clin N Am.

[60] Kagalwalla AF, Sentongo TA, Ritz S, Hess T, Nelson SP, Emerick KM, Melin-Aldana H (2006). Effect of six-food elimination diet on clinical and histologic outcomes in eosinophilic esophagitis. Clin Gastroenterol Hepatol.

[61] Gonsalves N, Ritz S, Yang G, Ditto A, Hirano I (2007). A prospective clinica trial of allergy testing and food elimination diet in adults with eosinophilic esophagitis (EE). Gastroenterology.

[62] Putnam PE, Rothenberg ME (2009). Eosinophilic esophagitis: concepts, controversies, and evidence. Curr Gastroenterol Rep.

[63] Nurko S, Teitelbaum J, Husain K, Buonomo C, Fox VL, Antonioli D, Fortunato C (2004). Association os Schatzki ring with eosinophilic esophagitis in children. J Pediatr Gastroenterol Nutr.

[64] Ngo P, Furuta GT, Antonioli DA, Fox VL (2006). Eosinophils in the esophagus—peptic or allergic eosinophilic esophagitis? Case series of three patients with esophageal eosinophilia. Am J Gastroenterol.

[65] Liacouras CA, Wenner WJ, Brown K, Ruchelli E (1998). Primary eosinophilic esophagitis in children: successful treatment with oral corticosteroids. J Pediatr Gastroenterol Nutr.

[66] King J, Khan S (2010). Eosinophilic esophagitis: perspectives of adult and pediatric gastroenterologists. Dig Dis Sci.

[67] Faubion WA, Perrault J, Burgart LJ, Zein NN, Clawson M, Freese DK (1998). Treatment of eosinophilic esophagitis with inhaled corticosteroids. J Pediatr Gastroenterol Nutr.

[68] Arora AS, Perrault J, Smyrk TC (2003). Topical corticosteroid treatment of dysphagia due to eosinophilic esophagitis in adults. Mayo Clin Proc.

[69] Langdon DE (2001). Fluticasone in eosinophilic corrugated ringed esophagus. Am J Gastroenterol.

[70] Martin-Munoz MF, Lucendo AJ, Navarro M, Letran A, Martin-Chavarri S, Burgos E, Martin-Esteban M (2006). Food allergies and eosinophilic esophagitis—two case studies. Digestion.

[71] Remedios M, Campbell C, Jones DM, Kerlin P (2006). Eosinophilic esophagitis in adults: clinical, endoscopic, histologic findings, and response to treatment with fluticasone propionate. Gastrointest Endosc.

[72] Perrault J, Arora AS, Clawson ML, Smyrk TC (2001). Treatment of eosinophilic esophagitis with steoid lavage in adult patients (abstract). Am J Gastroenterol.

[73] Attwood SE, Lewis CJ, Bronder CS, Morris CD, Armstrong GR, Whittam J (2003). Eosinophilic oesophagitis: a novel treatment using Montelukast. Gut.

[74] Gupta SK, Peters-Golden M, Fitzgerald JF, Croffie JM, Pfefferkorn MD, Molleston JP, Corkins MR (2006). Cysteinyl leukotriene levels in esophageal mucosal biopsies of children with eosinophilic inflammation: are they all the same?. Am J Gastroenterol.

[75] Netzer P, Gschossmann JM, Straumann A, Sendensky A, Weimann R, Schoepfer AM (2007). Corticosteroid-dependent eosinophilic oesophagitis: azathioprine and 6-mercaptopurine can induce and maintain long-term remission. Eur J Gastroenterol Hepatol.

[76] Kim YJ, Prussin C, Martin B, Law MA, Haverty TP, Nutman TB, Klion AD (2004). Rebound eosinophilia after treatment of hypereosinophilic syndrome and eosinophilic gastroenteritis with monoclonal anti-IL-5 antibody SCH55700. J Allergy Clin Immunol.

[77] Garrett JK, Jameson SC, Thomson B, Collins MH, Wagoner LE, Freese DK, Beck LA (2004). Anti-interleukin-5 (mepolizumab) therapy for hypereosinophilic syndromes. J Allergy Clin Immunol.

[78] Straumann A, Conus S, Grzonka P, Kita H, Kephart G, Bussmann C, Beglinger C Anti-interleukin-5 antibody treatment (mepolizumab) in active eosinophilic oesophagitis: a randomised, placebo-controlled, double-blind trial. Gut.

[79] Foroughi S, Foster B, Kim N, Bernardino LB, Scott LM, Hamilton RG, Metcalfe DD (2007). Anti-IgE treatment of eosinophil-associated gastrointestinal disorders. J Allergy Clin Immunol.

[80] Ford I, Murray H, Packard CJ, Shepherd J, Macfarlane PW, Cobbe SM (2007). Long-term follow-up of the West of Scotland Coronary Prevention Study. N Engl J Med.

[81] Straumann A, Bussmann C, Conus S, Beglinger C, Simon HU (2008). Anti-TNF-alpha (infliximab) therapy for severe adult eosinophilic esophagitis. J Allergy Clin Immunol.

[82] Prasad GA, Arora AS (2005). Spontaneous perforation in the ringed esophagus. Dis Esophagus.

[83] Cohen MS, Kaufman A, Dimarino AJ, Cohen S (2007). Eosinophilic esophagitis presenting as spontaneous esophageal rupture (Boerhaave's syndrome). Clin Gastroenterol Hepatol.

[84] Kaplan M, Mutlu EA, Jakate S, Bruninga K, Losurdo J, Keshavarzian A (2003). Endoscopy in eosinophilic esophagitis: "feline" esophagus and perforation risk. Clin Gastroenterol Hepatol.

[85] Straumann A, Bussmann C, Zuber M, Vannini S, Simon HU, Schoepfer A (2008). Eosinophilic esophagitis: analysis of food impaction and perforation in 251 adolescent and adult patients. Clin Gastroenterol Hepatol.

[86] Zuber-Jerger I, Ratiu N, Kullman F (2006). Long-lasting effect of endoscopic dilatation of an esophageal stenosis due to eosinophilic esophagitis. J Gastrointestin Liver Dis.

[87] Roberts-Thomson IC (2005). Images of interest. Gastrointestinal: eosinophilic esophagitis. J Gastroenterol Hepatol.

[88] Cohen MS, Kaufman AB, Palazzo JP, Nevin D, Dimarino AJ, Cohen S (2007). An audit of endoscopic complications in adult eosinophilic esophagitis. Clin Gastroenterol Hepatol.

[89] Rajagopalan J, Triadafilopoulos G (2009). Ring(s)-related esophageal meat bolus impaction: biopsy first, dilate later. Dis Esophagus.

[90] Eisenbach C, Merle U, Schirmacher P, Hansmann J, Stiehl A, Stremmel W, Kulaksiz H (2006). Perforation of the esophagus after dilation treatment for dysphagia in a patient with eosinophilic esophagitis. Endoscopy.

[91] Riou PJ, Nicholson AG, Pastorino U (1996). Esophageal rupture in a patient with idiopathic eosinophilic esophagitis. Ann Thorac Surg.

[92] Liguori G, Cortale M, Cimino F, Sozzi M (2008). Circumferential mucosal dissection and esophageal perforation in a patient with eosinophilic esophagitis. World J Gastroenterol.

[93] Schoepfer AM, Gonsalves N, Bussmann C, Conus S, Simon HU, Straumann A, Hirano I (2009). Esophageal Dilation in Eosinophilic Esophagitis: Effectiveness, Safety, and Impact on the Underlying Inflammation. Am J Gastroenterol.

[94] Pasha SF, DiBaise JK, Kim HJ, De Petris G, Crowell MD, Fleischer DE, Sharma VK (2007). Patient characteristics, clinical, endoscopic, and histologic findings in adult eosinophilic esophagitis: a case series and systematic review of the medical literature. Dis Esophagus.

[95] Lucendo AJ, De Rezende L (2007). Endoscopic dilation in eosinophilic esophagitis: a treatment strategy associated with a high risk of perforation. Endoscopy.

[96] Straumann A (2008). The natural history and complications of eosinophilic esophagitis. Gastrointest Endosc Clin N Am.

[97] Morrow JB, Vargo JJ, Goldblum JR, Richter JE (2001). The ringed esophagus: histological features of GERD. Am J Gastroenterol.

